# Effects of Perfluorooctanoic Acid on Metabolic Profiles in Brain and Liver of Mouse Revealed by a High-throughput Targeted Metabolomics Approach

**DOI:** 10.1038/srep23963

**Published:** 2016-04-01

**Authors:** Nanyang Yu, Si Wei, Meiying Li, Jingping Yang, Kan Li, Ling Jin, Yuwei Xie, John P. Giesy, Xiaowei Zhang, Hongxia Yu

**Affiliations:** 1 State Key Laboratory of Pollution Control and Resource Reuse, School of the Environment, Nanjing University, Nanjing, Jiangsu, People’s Republic of China; 2 Laboratory of Immunology and Reproductive Biology, School of Medicine, Nanjing University, Nanjing, Jiangsu, People’s Republic of China; 3 Department of Civil and Environmental Engineering, The Hong Kong Polytechnic University, Hung Hom, Kowloon, Hong Kong; 4 Department of Biomedical Veterinary Sciences and Toxicology Centre, University of Saskatchewan, Saskatoon, SK S7N 5B3, Canada; 5 School of Biology Sciences, University of Hong Kong, Hong Kong, SAR, China

## Abstract

Perfluorooctanoic acid (PFOA), a perfluoroalkyl acid, can result in hepatotoxicity and neurobehavioral effects in animals. The metabolome, which serves as a connection among transcriptome, proteome and toxic effects, provides pathway-based insights into effects of PFOA. Since understanding of changes in the metabolic profile during hepatotoxicity and neurotoxicity were still incomplete, a high-throughput targeted metabolomics approach (278 metabolites) was used to investigate effects of exposure to PFOA for 28 d on brain and liver of male Balb/c mice. Results of multivariate statistical analysis indicated that PFOA caused alterations in metabolic pathways in exposed individuals. Pathway analysis suggested that PFOA affected metabolism of amino acids, lipids, carbohydrates and energetics. Ten and 18 metabolites were identified as potential unique biomarkers of exposure to PFOA in brain and liver, respectively. In brain, PFOA affected concentrations of neurotransmitters, including serotonin, dopamine, norepinephrine, and glutamate in brain, which provides novel insights into mechanisms of PFOA-induced neurobehavioral effects. In liver, profiles of lipids revealed involvement of β-oxidation and biosynthesis of saturated and unsaturated fatty acids in PFOA-induced hepatotoxicity, while alterations in metabolism of arachidonic acid suggesting potential of PFOA to cause inflammation response in liver. These results provide insight into the mechanism and biomarkers for PFOA-induced effects.

Perfluorooctanoic acid (PFOA) is a member of an emerging class of contaminants known as perfluoroalkyl acids, which have been produced and used over 60 years^1^. PFOA was used as an aid in processing polymers, metal coatings (eg. non-stick cookware), surface coatings, and textile treatments^2^. During the lifetime of these products, PFOA can migrate from the products and thus enter the environment^3^. Also, historically much of the PFOA in the environment was released during manufacturing. The extremely strong carbon–fluorine bonds make PFOA stable in the environment^4^. Due to its extensive application, PFOA is ubiquitously in various environmental matrices, including drinking water^5^, food^6^, food packaging^7^, indoor dust^8^ and air^9^, which all constitute potential pathways for human exposure. As a result, PFOA has been detected in human serum^10^, cord blood^11^ and milk^12^. PFOA is not rapidly eliminated from the human body, with a half-life of 3.8 years in human blood^13^, thus raising the public concerns over the toxicological implications due to internal exposure.

Evidence has been emerging that exposure to PFOA can result in a range of adverse health effects, including toxicity during development and on the immune system and liver and on the neuro-endocrine systems of animals^14^. PFOA can also cause neurotoxicity in laboratory animals. Multiple neurobehavioral effects have been observed, including deranged spontaneous behavior, hyperactivity, and inversed effects on exploratory behavior in mice^15^,^16^, impaired imprinting behavior in chicken^17^, and disturbances in locomotion of zebrafish larvae^18^. Expression of CaMKII, GAP-43, synaptophysin, and tau in the hippocampus of the neonatal mouse were also affected^19^. Attention deficit hyperactivity disorder (ADHD) has been reported to be positively correlated with concentrations of PFOA in blood serum of children aged 12–15 in United States^20^, while no such associations were observed in a similar study conducted on Swedish subjects^21^. PFOA has been reported to cause adenomas of liver, testes and pancreas of rodents^22^ and delayed development of the mammary gland has been identified as a more sensitive endpoint^23^,^24^. Effects of PFOA on the liver can occur at lesser concentrations and have attracted more attention than some of the other effects. PFOA causes increased liver-to-body mass ratio, hepatocellular hypertrophy, hepatic triglyceride accumulation, peroxisomal β-oxidation, elevated serum liver enzymes, and lipid droplets in hepatic nuclei^14^,^25^,^26^,^27^,^28^. *In vitro*, PFOA can activate multiple nuclear receptors, such as peroxisome proliferator-activated receptor alpha (PPARα), constitutive androstane receptor (CAR), pregnane X receptor (PXR), and estrogen receptor (ER)^29^. These studies focused on one or two hepatic effects of PFOA so it was deemed appropriate to investigate effects of PFOA on the liver by use of a comprehensive method.

Metabolomics measures smaller-molecular-mass metabolites by either nuclear magnetic resonance (NMR) spectroscopy or mass spectrometry, and is an indicator of changes in pathways of metabolism, which are end points of expression of genes and actions of functional proteins. Metabolomics can further understanding of toxicant-induced responses and discover biomarkers, and has been recognized as a promising tool for monitoring of changes caused by stressors^30^. Although several transcriptomic and proteomic studies^31^,^32^,^33^,^34^,^35^ have been conducted on perfluoroalkyl acids (PFAAs), including PFOA, few studies have used metabolomics to elucidate PFOA-induced toxicology. These studies focused on effects on metabolites in earthworm^36^ and L-02 cells^37^. However, the contribution of the results of these studies to the understanding of mechanisms of effects of PFOA on mammals is limited and there are no *in vivo* studies of metabolomics of mammals. In this study, a high-throughput targeted metabolomics approach was used to investigate effects of PFOA on profiles of metabolites in liver and brain of mice. By analyzing changes of functionally important endogenous metabolites involved in various biochemical pathways in liver and brain, mechanisms of PFOA-induced hepatotoxicity and neurotoxicity were elucidated and potential biomarkers for exposure to PFOA were developed.

## Results and Discussion

### Concentrations of PFOA and its effect on body and organs

After a 28-day exposure to PFOA, concentrations of PFOA in brain, blood, and liver of mice in the two groups exposed to PFOA (greater-dose: 2.5 mg/kg/day; lesser-dose: 0.5 mg/kg/day) were significantly greater than those in the respective tissues of mice from the unexposed control group (Table 1). Due to control by the blood-brain barrier, concentrations of PFOA in brain were approximately 100-fold less than that in blood or liver. Mean concentrations of PFOA in serum of mice in the lesser dose group was 29.34 μg PFOA/mL, which was similar to concentrations of PFOA in blood serum of persons occupationally exposed to PFOA^38^. Exposure to PFOA for 28 d resulted in greater masses of liver and ratios of liver-to-body mass than those in controls. Masses of liver and the liver-to-body mass ratio of only the high-dose group (2.5 mg/kg/day) were significantly (p < 0.05, one-way ANOVA with Tukey post hoc comparisons) greater than those of the control group. The doses of PFOA were based on previous studies^39^,^40^,^41^ involving similar exposure regimes that resulted in a range of toxicological consequences, including body weight loss, liver enlargement, serum alanine aminotransferase increase, insulin hypersensitivity.

We maintained strict QA/QC procedures to minimize the background contamination. For example, a PFOA isolator column was placed in-line between the solvent mixer and the injector in the LC-MS/MS instrument to reduce the background PFOA from Teflon tubing. Consequently, the concentrations of PFOA in the procedural blanks were below its method LOQ (1 ng/mL). We still detected a trace level of PFOA in the control mouse group, which is, however, far below the final internal concentrations in the low-dose and high-dose exposed groups. Therefore, we reasoned that the experimental mice may have had prior exposure to contaminated food and water containing a low level of PFOA before the current exposure study.

### PCA and PLS-DA for profiles of metabolites and precursors in brain and liver

Amino acids, acylcarnitines (free carnitine, acylcarnitines (AC Cx:y), hydroxylacylcarnitines (AC C(OH)x:y), and dicarboxylacylcarnitines (AC Cx:y-DC)), sphingomyelins (sphingomyelins (SMx:y) and N-hydroxylacyloylsphingosyl-phosphocholines (SM(OH)x:y)), phosphatidylcholines (phosphatidylcholines (PC aa Cx:y), plasmalogen/plasmenogen phosphatidylcholines (PC ae Cx:y) and lyso-phosphatidylcholines (lysoPC a Cx:y)), and biogenic amines were quantified in both brain and liver. Lipid chain composition is abbreviated as Cx:y, where x is the number of carbon atoms and y is the number of double bonds. In addition, neurotransmitters were quantified in brain, and fatty acids, oxidized polyunsaturated fatty acids, and intermediates of energy metabolism were quantified in liver. In total, 193 metabolites in brain and 274 metabolites in liver were quantified. By mapping these metabolites to their respective metabolic pathways by use of the Kyoto Encyclopedia of Genes and Genomes (KEGG), it was determined that inferences about metabolism pathways involving amino acids, carbohydrates, lipids and energy could be made (see Supplementary Fig. S1).

**Figure 1:**
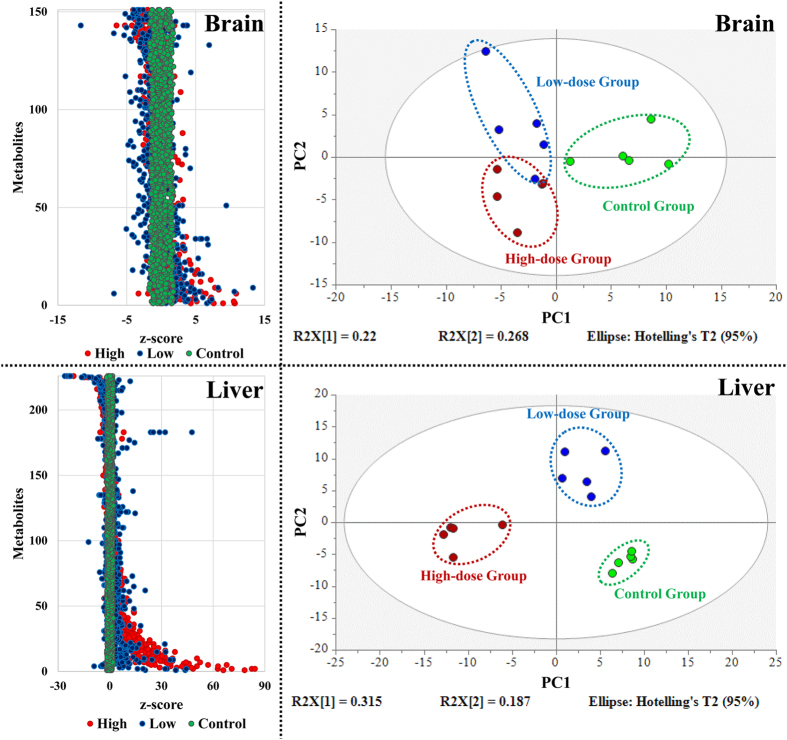
Z-score plots for each metabolites in brain and liver, and PLS-DA score plots on the metabolic profiles in brain and liver (red: high-dose group; blue: low-dose group; green: control group).

For brain, 43 out of 193 metabolites (22.3%) were observed in less than 20% of samples. For liver, 48 out of 274 metabolites (17.5%) were observed in less than 20% of samples. Profiles of metabolites in brain and liver were displayed as a plot of z-scores (Fig. 1). The z-scores represent deviations of concentrations of metabolites from the control group. In samples of brain ranges of z-scores of the low-dose and high-dose group were −11.7 to 17.2 and −6.5 to 10.9, respectively. While for liver, ranges of z-scores in the low-dose group and the high-dose group were −26.1 to 185 and −25.0 to 110, respectively. To further examine changes in patterns of metabolites caused by PFOA, multivariate pattern recognition analysis was performed on normalized data. To reduce the effect of magnitudes of absolute concentrations of metabolites and improve interpretability, all pattern recognition techniques were applied to unit variance scaled data^42^.

PCA analysis was conducted to identify outliers and make an initial discrimination among groups. Plots of PCA scores are provided in the Supplementary Information (see Supplementary Fig. S2). In the PCA score plots based on the first two components, all data points were located within 95% confidence ellipses and no outliers were found for any samples of liver or brain. For liver samples, three groups were clearly separated in plots of PCA scores based on the first two components. For brain PFOA-exposure groups were separated from the control group based on the first three components. A supervised PLS-DA was then performed to further investigate differences among groups.

In brain, the analysis obtained a PLS-DA model with five components, characterized by a faithful representation of the data (R^2^Y = 95.0%) and a good cumulative predictive capacity (Q^2^ = 0.800). The score plot of PLS-DA based on the first two components showed that the two groups exposed to PFOA clustered together and were separated from the control group along the PC1 axis (Fig. 1). Along the PC2 axis, the low-dose group and the high-dose groups were separated but not completely. When 200 permutations were run, the PLS-DA model had a larger Q^2^ value that suggested separation among the three groups was statistically significant (see Supplementary Fig. S3). Thus, it can be concluded that both doses of PFOA used in this study resulted in alterations of the patterns of metabolites in brain.

In liver, the PLS-DA model obtained had only two components. Compared with the PLS-DA model for brain, the model for liver had a less faithful representation of the data (R^2^Y = 91.8%) but a better cumulative predictive capacity (Q^2^ = 0.843). The score plot of PLS-DA based on the first two components showed that all three groups were completely separated from each other along both the PC1 PC2 axes (Fig. 1). Based the results of 200 permutation tests, the model had a larger Q^2^ value than those for permutation tests, which suggested separation among the three groups was statistically significant (see Supplementary Fig. S3). Thus, separation of samples of liver (Fig. 1) was statistically significant. These results confirmed that the PFOA caused alterations in pathways represented by the relative concentrations of metabolites in liver. Compared with the results for brain, those for liver exhibited better separation among groups, especially for the two PFOA-exposure groups. This difference between liver and brain could be due to the large differences between doses received by these two tissues (Table 1).

### Differential patterns of metabolites and potential biomarkers in PFOA-induced metabolic alteration

**Figure 2:**
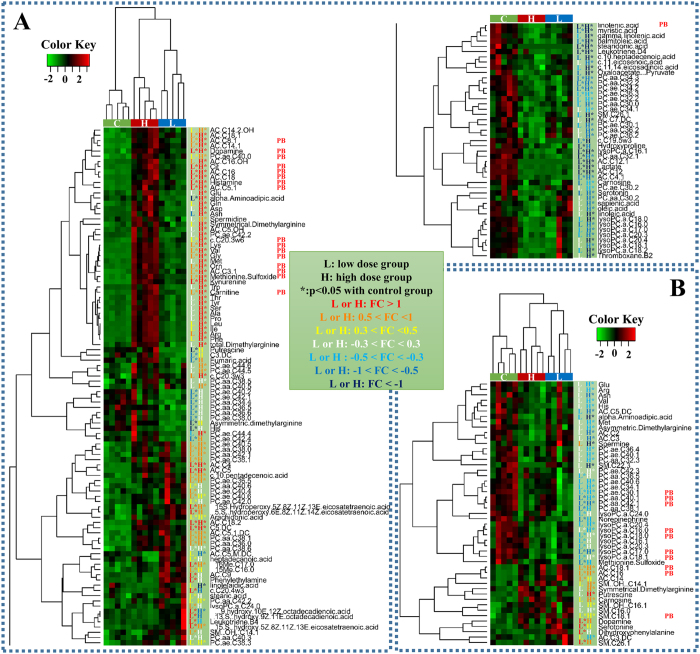
Heat maps produced by clustering of the differential metabolites and samples in liver (**A**) and brain (**B**) with fold change (FC) for low-dose group and high-dose group. PB for the potential biomarker.

In brain, a total of 49 out of 151 metabolites and substrates were identified as being different (p < 0.05, q < 0.14) between the two groups exposed to PFOA (Fig. 2B). A total of 49 differentially expressed metabolites were clustered according to the change of metabolite concentration in three groups (Fig. 2B). Compared with the control group, 10 metabolites were significantly different from control values in both the low- and high-dose groups and had similar directional trends with concentrations of three metabolites being greater than in the controls and seven being less. These metabolites were identified as potential biomarkers of functional responses of brain to exposure to PFOA (Fig. 2B and Supplementary Table S1). However, there was no statistically significant difference for these metabolites between the two PFOA-exposed groups. All 10 metabolites including 2 acylcarnitines, 1 sphingomyelins, and 7 phosphatidylcholines were involved in lipid metabolism, so this pattern implied that metabolites involved in lipid metabolism were more sensitive to the effects of exposure to PFOA than were other metabolites.

For liver a total of 144 of 226 metabolites investigated were identified as being different (p < 0.05, q < 0.033) between the two PFOA exposure groups and the controls (Fig. 2A). The 144 differential metabolites were clustered according to the change of metabolite concentration in three groups. Among these metabolites, 18 were found to be statistically significant difference in both two PFOA exposure groups. The trends in direction of changes were consistent between the two groups with concentrations of 17 being greater in the two treatments relative to that in the controls and the concentration of one metabolite being less in the treatments than in the controls. Therefore, these 18 metabolites were identified as potential biomarkers of functional responses of live to exposure to PFOA (Fig. 2A and Supplementary Table S1). These metabolites, substrates and precursors included 5 amino acids, 4 biogenic amines, 6 acylcarnitines, 1 phosphatidylcholines, and 2 polyunsaturated fatty acids, and were mainly involved in amino acid metabolism and lipid metabolism.

### Analyses of PFOA-induced alterations in metabolic pathways

**Figure 3:**
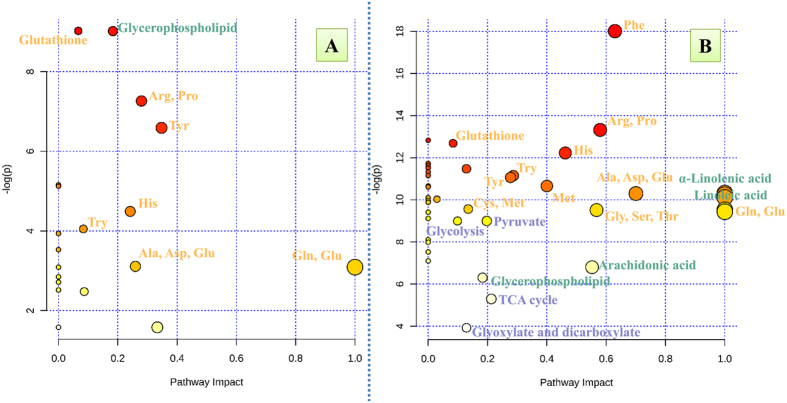
Metabolic pathway analysis. The important metabolic pathway influenced by PFOA for brain (**A**) and liver (**B**) with pathway enrichment analysis in MetaboAnalyst, yellow: amino acid metabolism; green: lipid metabolism; purple: carbohydrate metabolism and energy metabolism.

Based on the differential metabolites, relevant metabolic pathways affected by PFOA in liver and brain were further investigated to determine which pathways were affected. When differential metabolites in brain were investigated by use of the KEGG pathway database, it was found that metabolisms of both amino acids and lipids were affected by exposure to PFOA (see Supplementary Fig. S4). By use of pathway enrichment analysis in MetaboAnalyst, it was determined that metabolism of glutathione, glycerophospholipid, arginine, proline, tyrosine, histidine, tryptophan, alanine, aspartate, glutamate, and D-glutamine and D-glutamate were involved in pathways perturbed by exposure to PFOA in brain (p < 0.05, Fig. 3A).

In liver, pathways involving amino acids, lipids, carbohydrates, and energy metabolism were influenced by PFOA (see Supplementary Fig. S4). During pathway enrichment analysis in MetaboAnalyst, it was further found that in liver metabolism involving phenylalanine, arginine, proline, glutathione, histidine, tryptophan, tyrosine, methane, alanine, aspartate, glutamate, α-linolenic acid, cysteine, methionine, linoleic acid, glycine, serine, threonine, D-glutamine, D-glutamate, pyruvate, glycolysis, arachidonic acid, glycerophospholipid, the TCA cycle, glyoxylate and dicarboxylate were altered by exposure to PFOA (p < 0.05, Fig. 3B).

### Neurotransmitters in brain

PFOA affected metabolism involving several amino acids in brain. It is known that metabolism involving amino acids an important part of the processing of neurotransmitters. In this study, concentrations of four neurotransmitters were significantly altered by exposure to PFOA. In the low-dose group, concentrations of serotonin and dopamine were greater than those in controls while concentrations of norepinephrine were less than those in controls. In the high-dose group, concentrations of glutamate were less than those in controls. Although the four neurotransmitters were not identified as differential metabolites in both PFOA-exposure groups, they exhibited similar trends in the two groups exposed to PFOA (see Supplementary Fig. S5).

Concentrations of neurotransmitters in brain can affect activation of neurons, which is important during transmission of signals from one neuron to another. Therefore, neurobehavioral effects may be associated with the level of neurotransmitters. Deranged spontaneous behavior, hyperactivity, and adverse effects on exploratory behavior have been demonstrated in mice exposed to PFOA^15^,^16^. Although neurobehavioral effects following prenatal or neonatal exposure were the focus of those studies, PFOA-induced neuroendocrine effect might also be occur in those exposed mice. It can be speculated that the deranged spontaneous behavior and hyperactivity might be consequences of greater concentrations of dopamine, which can affect the pleasure behavior^43^ and is a key component of neurobiology associated with aggressive behavior^44^, and that the adverse effects on exploratory behavior may result from the lesser concentrations of glutamate, which can affect synaptic plasticity and memory^45^. It has been reported that concentrations of PFOS was greater in brains of rat fetuses compared with the brains of their respective dams and juveniles^46^. This result suggests that at least some PFAAs can more easily pass through the blood brain barrier of rat fetuses. Therefore, it is postulated that alteration of neurotransmitters in brain might be more important biological effects during early development of mammals.

### Alterations of metabolites in lipid metabolism during PFOA exposure

Most of the potential biomarkers in brain (10 metabolites) and liver (9 metabolites) belong to the lipid metabolic pathway. This results suggests that metabolism of lipids was more sensitive effects of PFOA and that alterations in profiles of relative concentrations of fatty acids and lipids (acylcarnitines, sphingomyelins, phosphatidylcholines, and oxidized polyunsaturated fatty acids) were major effects of exposure to PFOA.

Acylcarnitine congeners are essential for metabolism of lipids and are involved primarily in β-oxidation of fatty acids. Potential biomarkers of exposure including AC C16 and AC C18:1 were greater in brain, and carnitine, AC C18, AC C16, AC C8:1, AC C5:1, and AC C3:1 were greater in liver of mice exposed to PFOA. These results of this study are reported here are consistent with a study of effects of PFOA on L-02, human, liver cells, which also found to have greater concentrations of acylcarnitine congeners after exposure to PFOA^37^. The difference between the two studies was that in our study not only the shorter-chain but also longer-chain acylcarnitine congeners were upregulated in liver. Based on results for brain, it is postulated that longer chain acylcarnitine congeners were more sensitive to the tissue dose of PFOA. This result suggests that PFOA can induce β-oxidation of fatty acids in brain and liver.

All differential phosphatidylcholines, which were involved in metabolism of glycerophospholipids, were less in brains of mice exposed to PFOA (Fig. 2B). Phosphatidylcholines serve as reservoirs for choline, which is the precursor of acetylcholine, which is the primary neurotransmitter for cholinergic neurons^47^. It has been found previously that susceptibility of the cholinergic system in adult mice was affected by neonatal exposure to PFOA^24^. It can be postulated that lesser concentrations of phosphatidylcholines in brain could be associated with effects of PFOA on the cholinergic system.

There was no a consistent trend for differential concentrations of phosphatidylcholines in liver (Fig. 2A). However, exposure to PFOA affected the profile of phosphatidylcholines in liver, especially for plasmalogen/plasmenogen phosphatidylcholines (see Supplementary Fig. S6). There was a statistically significant difference (p < 0.05, Mann-Whitney U test) in the profile of phosphatidylcholines between the exposed groups and the control group. The proportion of phosphatidylcholines with more carbons and more double bonds was greater while that of phosphatidylcholines with fewer carbons and fewer double bonds were less in mice exposed to PFOA. Similar trends were observed for the profile of fatty acids profile (see Supplementary Fig. S6). A change to fatty acids with more carbon atoms and double bonds is the result of biosynthesis reactions of saturated and unsaturated fatty acids^48^. From these results it can be concluded that exposure to PFOA might stimulate biosynthesis of fatty acids, thus altering the profile of phosphatidylcholines and fatty acids in liver.

In previous studies of transcriptomic and proteomics, it has been found that β-oxidation of fatty acids and biosynthesis of fatty acids were both significantly stimulated by PFOA^49^, perfluorononanoic acid (PFNA)^34^ and perfluorododecanoic acid (PFDoDA)^31^ in rodent liver. The metabolomics data reported here also implied that PFOA could stimulate catabolism and anabolism of lipids in liver. This could be a reason for the different trend of phosphatidylcholines and fatty acids in liver of PFOA-exposure groups (Fig. 2A). Considering the consistent trend for phosphatidylcholines (Fig. 2A) and the tissue dose (Table 1) in brain of PFOA-exposure groups, it can be postulated that catabolism of lipids could be more sensitive to PFOA than was their anabolism. Likely the previous proteomic study of PFNA^34^, PFOA could also activate different transcription factors to regulate lipid catabolism (PPARα) and anabolism (sterol regulatory element-binding proteins)^34^.

In liver, except for metabolism of glycerophospholipid, polyunsaturated fatty acids metabolism (α-linolenic acid, linoleic acid, and arachidonic acid, Fig. 3B) was also affected by exposure to PFOA. Arachidonic acid is a precursor in production of prostaglandins, thrombaxanes, and leukotrienes, which are mediators in inflammation^50^. In previous studies of transcriptomic and proteomic effects of PFNA^34^, PFDoDA^31^, and PFOS^35^, the Cyp4a family, which is regulated by PPARα, and involved in ω-hydroxylation of fatty acids and arachidonic acid metabolism in liver, was upregulated in rodent. The authors of that paper suggested the basis of disturbance of arachidonic acid metabolism. In this study, prostaglandins (PGD2, PGE2, and PGF2α), which can cause vasodilation, fever, and pain^50^, were upregulated slightly but without a statistical significance (see Supplementary Fig. S7). The ratio of TXB2/6-keto-PGF1α, which indicates the ratio of TXA2 and PGI2, was significantly less (see Supplementary Fig. S6). This result indicated vasodilation of microvasculature, lessened adherent leukocytes, and improved flow velocity in liver, which could result in ischemic injury to the liver^51^. For leukotrienes, LTD4 was significantly less in mice exposed to PFOA. In contrast, LTB4 was significantly greater in the low-dose group but less in the high-dose group (see Supplementary Fig. S7). These results provide evidence for the involvement of inflammation during PFOA-induced hepatotoxicity, which are complementary to an increasing dose-response relationship for histamine (Fig. 2A), a mediator in inflammation^52^.

## Conclusions

Changes in the metabolic profiles of liver and brain of mice exposed to PFOA were quantified by a high-throughput target metabolomics approach and offered a connection among transcriptome, proteome and PFOA-induced hepatomegaly and neurobehavioral effects. Metabolisms of amino acids, lipids, carbohydrates, and energy metabolism were involved in PFOA-induce metabolic disturbances of both brain and liver, and metabolism of lipids was more sensitive to effects of PFOA. Profiles of lipids in mice exposed to PFOA suggested that β-oxidation and biosynthesis of fatty acids and inflammation were involved in PFOA-induced hepatomegaly. PFOA-induced neuroendocrine effects, such as alterations of neurotransmitters in brain provides a mechanistic basis for PFOA-induced neurobehavioral effects, which can provide insights on the molecular mechanism of neurotoxicity. Certainly, these PFOA-induced effects, especially PFOA-induced neuroendocrine effects, were necessary to be further confirmed with more analysis (RT-PCR and western-blot). In addition, concentrations of PFOA in blood serum in mice exposed to the lesser concentration of PFOA were similar to concentrations of PFOA in blood of occupational exposed humans. Thus the results of this study could be relevant to these greater concentrations and PFOA-induced metabolic effects observed in this study could be used as measurement endpoints in assessment of risks of PFOA to humans and could suggest potential functional biomarkers of exposure to PFOA. Certainly, additional work will be necessary to confirm the conclusion.

## Methods and Materials

### Maintenance of mice, exposure to PFOA and collection of samples

The experimental protocols were approved by School of the Environment, Nanjing University, and all experiments were carried out in accordance with the approved guidelines. Six-week-old male Balb/c mice obtained from the Experimental Animal Center, Academy of Military Medical Science, Beijing, China, were maintained under conditions of temperature- (22 ± 2 °C) and light-controlled (12 hr light/dark cycle) room. Mice were randomly divided into three groups (n = 5 each): high-dose (2.5 mg PFOA/kg body weight/day), low-dose (0.5 mg PFOA/kg body weight/day), and control group, and housed in 3 polypropylene cages. The control group was administered 0.1 mL of corn oil daily. The exposed groups received a 28-day consecutive administration of PFOA in corn oil (0.1 mL) via oral infusion. To minimize background contamination arising from PFC-contained bottles, polypropylene bottles were used for commercial chow and Milli-Q water. After 28-day exposure, each mouse was euthanized and samples were collected from liver and brain. The samples were then weighed and rinsed with phosphate-buffered saline (PBS), and stored at −80 °C until analysis.

### Sample preparation and quantification of metabolites

Samples of frozen tissue were homogenized in extraction buffer (3 μl extraction buffer/mg tissue) using Precellys® beads, then an aliquot of each sample was centrifuged. The supernatant was collected for quantification of metabolites. Samples were randomized on a 96-well plate prior to analysis to avoid potential interactions between samples and order of injection. Concentrations of metabolites in extracts of brain or liver were determined by Biocrates Life Sciences (Innsbruck, Austria). All pre-analytical and analytical procedures were performed, documented and reviewed according to ISO 9001:2008 certified in-house quality management rules and guidelines.

AbsoluteIDQ^TM^ p180 kits (Biocrates life sciences, Innsbruck, Austria) were used for quantification of amino acids, acylcarnitines, and dicarboxylacylcarnitines, sphingomyelins, phosphatidylcholines, and biogenic amines. Except for fatty acids, all metabolites were determined by use of internal standards by an AB SCIEX 4000 Qtrap^TM^ mass spectrometer (AB Sciex, Darmstadt, Germany) with electrospray ionization. Prior to analysis, amino acids, biogenic amines and neurotrasmitters were derivatized by use of phenylisothiocyanate with internal standards. Acylcarnitines, sphingomyelins, phosphatidylcholines were quantified by use of a flow injection analysis-tandem mass spectrometry method. Other metabolites were identified and quantified by use of LC-MS or LC-MS/MS method. After derivatization, fatty acids were determined as their corresponding methyl ester derivatives by separation by use of gas chromatography coupled with mass spectrometric detection (Agilent 7890GC/5975 MSD). External standard calibration curves were used to calculate the concentrations of fatty acids. A detail summary of metabolites analysis was shown in Supplementary Methods.

### Quantification of PFOA in liver, brain and blood

Quantification of PFOA was accomplished by use of previously described methods^53^. A 10-mg sample of liver or brain was homogenized in 100 μL Milli-Q water. For blood, 10 μL of samples were diluted in 90 μL Milli-Q water. Then 10 ng of mass-labeled internal standard, ^13^C_4_-PFOA was added to, 200 μL of sodium carbonate buffer (0.25 M, pH 10), and 100 μL tetrabutyl ammonium hydrogen sulfate (TBAHS, 0.5 M) solution were added. After mixing, samples were extracted with 0.5 mL of MTBE by shaking for 20 min, the MTBE layer separated by centrifugation. The extraction was repeated twice. MTBE layers were combined and evaporated to dryness under nitrogen and then reconstituted in 100 μL of methanol. The concentrate was passed through a polypropylene-membrane syringe filter (4 mm, 0.2 μm, Thermo Scientific). PFOA was analyzed by high performance liquid chromatography (Agilent 1260 Infinity LC, Agilent Technologies) tandem mass spectrometry (API 4000, AB Sciex, Darmstadt, Germany). Details of instrument parameters and QA/QC can be found in Supplementary Methods.

### Data processing and analysis

Metabolites with a detected frequency < 20% were excluded from further data analysis. For metabolites less than the limit of detection (LOD), a value of half of the LOD was assigned as a surrogate value to minimize biases introduced by including a value of zero. Concentrations of each metabolite were normalized to masses of samples. Samples from the control group were assigned as a reference distribution, and z-scores were calculated for each metabolite. For each sample from exposed groups, concentrations of metabolites were centered by the mean of each metabolite and scaled by the standard deviation of the corresponding metabolite in the control group to obtain the z-score. Fold change (FC) for each metabolite in the two PFOA-exposed groups was calculated. Normalized data was imported into SIMCA-P (Umetrics, Sweden) for multivariate statistical analyses. Outliers were identified and excluded by use of principal component analysis (PCA). Partial least-squares-discriminant analysis (PLS-DA) was performed to construct a relationship between groups and metabolic profiles. To verify the robustness, predictive power, and validity of the PLS-DA model, seven-fold cross-validation, Q^2^ parameter, and 200 permutation tests were performed. To investigate differences of concentrations of metabolites among groups, the Mann-Whitney U test was applied to normalized data by use of SPSS Statistics (SPSS Inc., Chicago, IL). Positive false discovery rates (pFDR) were calculated by use of the p-value in R package “q-value”^54^. The relative concentrations of metabolites in the three treatments are shown by use of heat maps based on R statistical package. MetaboAnalyst 2.0^55^ and KEGG were used to identify the biochemical pathways affected by PFOA exposure. Log P was the logarithm of p value from the pathway enrichment analysis. Log P reflected the deviation of metabolic pathways between the two groups based on the concentration of metabolites. Pathway impact reflected the deviation of metabolic pathways between the two groups based on the positions of metabolites.

## Additional Information

**How to cite this article**: Yu, N. *et al.* Effects of Perfluorooctanoic Acid on Metabolic Profiles in Brain and Liver of Mouse Revealed by a High-throughput Targeted Metabolomics Approach. *Sci. Rep.* **6**, 23963; doi: 10.1038/srep23963 (2016).

## Supplementary Material

Supplementary Information

## Figures and Tables

**Table 1:** PFOA concentration, body masses, and organ indexes after 28 d exposure to PFOA.

Group	Control	Low	High
Dose of PFOA (mg/kg/day)	0	0.5	2.5
PFOA in blood (μg/mL)	0.011(0.0057)	29.34(5.06)**	114.3(21.80)**
PFOA in brain (μg/g)	0.011(0.0051)	0.26(0.062)**	0.92(0.12)**
PFOA in liver (μg/g)	0.026(0.021)	29.50(6.44)**	166.9(63.83)**
Body(g)	21.08(1.44)	20.58(2.39)	20.70(1.07)
Liver(g)	1.08(0.13)	1.14(0.17)	1.37(0.21)*
Liver/Body(%)	5.10(0.41)	5.52(0.27)	6.62(0.66)**
Brain(g)	0.52(0.05)	0.52(0.07)	0.43(0.07)
Brain/Body(%)	2.49(0.28)	2.53(0.38)	2.06(0.32)

Data are mean (standard deviation).

*P < 0.05, **P < 0.01 versus their corresponding controls, using one-way ANOVA with Tukey post hoc comparison.
