# Supplementary material for: Effects of Perfluorooctanoic Acid on Metabolic Profiles in Brain and Liver of Mouse Revealed by a High-throughput Targeted Metabolomics Approach
Source: Sci Rep. 2016 Apr 1;6:23963. doi: 10.1038/srep23963 (PMC4817033; doi:10.1038/srep23963)
Supplement: Supplementary Information [file srep23963-s1.pdf]

## Supplementary Information

# Effects of Perfluorooctanoic Acid on Metabolic Profiles in Brain and Liver of Mouse by a High-throughput Targeted Metabolomics Approach

Nanyang Yu[†], Si Wei[†,*], Meiying Li[†], Jingping Yang[‡], Kan Li[†], Ling Jin[§], Yuwei Xie[†], John P. Giesy[†,⊥,//], Xiaowei Zhang, Hongxia Yu[†,*]

[†]State Key Laboratory of Pollution Control and Resource Reuse, School of the Environment, Nanjing University, Nanjing, Jiangsu, People's Republic of China

[‡]Laboratory of Immunology and Reproductive Biology, School of Medicine, Nanjing University, Nanjing, Jiangsu, People's Republic of China

[§]Department of Civil and Environmental Engineering, The Hong Kong Polytechnic University, Hung Hom, Kowloon, Hong Kong

[⊥]Department of Biomedical Veterinary Sciences and Toxicology Centre, University of Saskatchewan, Saskatoon, SK S7N 5B3, Canada

[//]School of Biology Sciences, University of Hong Kong, Hong Kong, SAR, China

**Corresponding author:**

Name: Dr. Si Wei

Phone: +86 25 8968 0356; fax: +86 25 8968 0356; e-mail: weisi@nju.edu.cn

Name: Dr. Hongxia Yu

Phone: +86 25 8968 0356; fax: +86 25 8968 0356; e-mail: yuhx@nju.edu.cn

| | |
|---|---|
| **Supplementary Methods** | **P.S3** |
| **Supplementary Table S1** | **P.S6** |
| **Supplementary Figure S1.** | **P.S8** |
| **Supplementary Figure S2.** | **P.S9** |
| **Supplementary Figure S3.** | **P.S10** |
| **Supplementary Figure S4.** | **P.S11** |
| **Supplementary Figure S5.** | **P.S12** |
| **Supplementary Figure S6.** | **P.S13** |
| **Supplementary Figure S7.** | **P.S14** |

## Supplementary Methods

### Metabolite analysis

**AbsoluteIDQ™ p180 kit assay.** Biocrates' commercially available KIT plates were used for quantification of amino acids, acylcarnitines, sphingomyelins, phosphatidylcholines, hexoses, and biogenic amines. The fully automated assay was based on phenylisothiocyanate (PITC) derivatization in the presence of internal standards followed by flow injection analysis-tandem mass spectrometry (FIA-MS/MS) (acylcarnitines, lipids, and hexose) and LC/MS (amino acids, biogenic amines) using an AB SCIEX 4000 QTrap™ mass spectrometer (AB SCIEX, Darmstadt, Germany) with electrospray ionization. The experimental metabolomics measurement technique was described in detail by patent US 2007/0004044.

**Oxidized polyunsaturated fatty acids.** Eicosanoids and other oxidized polyunsaturated fatty acids were extracted from samples with aqueous acetonitrile that contained deuterated internal standards. The metabolites were determined by LC-MS/MS with Multiple Reaction Monitoring (MRM) in negative mode using a SCIEX API 4000 QTrap mass spectrometer with electrospray ionization.

**Intermediates of the energy metabolism.** For quantification of energy metabolism intermediates (glycolysis, citrate cycle, pentose phosphate pathway, urea cycle) hydrophilic interaction liquid chromatography (HILIC)-ESI-MS/MS in highly selective negative MRM detection mode was used. The MRM detection was performed using a SCIEX 4000 QTrap™ tandem mass spectrometry instrument (Applied Biosystems/MDS Analytical Technologies). Protein was precipitated and extracted simultaneously with aqueous methanol in a 96 well plate format. Internal standards (ratio external to

internal standard) and external calibration were used for quantitation.

**Free fatty acids.** Concentrations of individual free fatty acids in liver and urine samples from mice were quantified as their corresponding methyl ester derivatives (FAME's) using gas chromatography coupled with mass spectrometric detection (Agilent 7890 GC / 5975 MSD) after derivatization. Samples were treated with methanolic HCl for a prolonged time period to completely convert both free fatty acids into their methyl esters representing the free fatty acid (FFA) content. Chromatograms in Selected Ion Monitoring (SIM) mode with four characteristic ions were recorded for quantitation of individual FAME's. External standard calibration curves were used to calculate the corresponding concentrations. Unknown FAME's (external standards not available) were identified using a combination of: spectra recorded in SCAN mode, respective ratios of characteristic ions and the retention behavior. Their (semi)-quantification was carried out with response factors extra- and/or interpolated from the nearby eluting compounds having the same number of double bonds.

**Neurotransmitter.** Quantification of neurotransmitters was carried out in a 96 well plate format and based on a PITC (phenylisothiocyanate)-derivatization in the presence of internal standards followed by LC-ESI-MS/MS in selective multiple reaction monitoring (MRM) mode using an AB Sciex 4000 QTrap™mass spectrometer (AB Sciex, Darmstadt, Germany). 5-HIAA and its corresponding internal standard cannot be derivatized with PITC because they do not contain an aliphatic amino group like the other 6 neurotransmitters. Therefore, the chromatographic peak of the un-derivatized 5-HIAA was slightly broader and the detection in the mass spectrometer was less sensitive (greater LOD) compared to the other derivatized neurotransmitters. Apart from that, 5-HIAA can be quantified without

restrictions.

**Instrument analysis on PFOA and QA/QC.**

PFOA was analyzed by high performance liquid chromatography (Agilent 1260 Infinity LC, Agilent Technologies) tandem mass spectrometry (API 4000, AB Sciex, Darmstadt, Germany) with an ACQUITY BEH C18 column (2.1 mm × 50 mm, 2.5 μm, Waters, Milford, MA, USA). The column temperature was held at 40 ℃. The mobile phases were 2 mM ammonium acetate in water (A) and methanol (B). The sample injection volume was 5 μL. The HPLC gradient program was optimized as follows: the flow rate was set at 400 μL/min with starting at 5 % of solvent B held until 0.50 min, increasing to 20 % of solvent B until 1.5 min, to 50 % of solvent B until 5 min, to 65 % of solvent B until 13 min, to 85 % of solvent B until 14.5 min, further 100 % of solvent B until 15.5 min, and then keeping 5 % of solvent B until 18 min for equilibration. The mass spectrometer was operated in negative electrospray ionization multiple reaction monitoring (MRM) mode (413.1-368.8 and 413.1-168.9 for PFOA, 417-372 for $^{13}C_4$-PFOA).

Quantification of PFOA was performed by use of an internal standard calibration curve ($r > 0.99$). The limit of quantification (LOQ) for instrument was defined as the minimum point on the calibration curve that could be accurately measured within ± 20 % of its theoretical value. PFOA in procedural blanks were below its method LOQ (1 ng/mL). Mean matrix spike recoveries of PFOA (20 ng/g or 20 ng/mL) in blood (n = 3), brain (n = 3), and liver (n = 3) were 79.8±1.6%, 81.2±2.5% and 78.1±1.4%, respectively.

**Supplementary Table S1. Potential biomarkers for exposure to PFOA.**

| Biomarker | Metabolism | Low dose group | | | | High dose group | | | | Concentration(pmol/mg wet) | | |
|---|---|---|---|---|---|---|---|---|---|---|---|---|
| | | FC[a] | p | q | | FC[a] | p | q | | Control | Low | High |
| **Brain** | | | | | | | | | | | | |
| AC C16 | Lipid, β-oxidation | 0.65 | 0.028 | 0.118 | ↑ | 0.60 | 0.047 | 0.057 | ↑ | 0.64 | 1.01 | 0.97 |
| AC C18:1 | Lipid, β-oxidation | 0.51 | 0.028 | 0.118 | ↑ | 0.58 | 0.047 | 0.057 | ↑ | 0.31 | 0.44 | 0.46 |
| SM C18:1 | Lipid, phospholipid | 0.47 | 0.047 | 0.143 | ↑ | 0.41 | 0.047 | 0.057 | ↑ | 7.52 | 10.4 | 9.96 |
| lysoPC a C16:0 | Lipid, phospholipid | -0.30 | 0.047 | 0.143 | ↓ | -0.35 | 0.028 | 0.048 | ↓ | 192 | 156 | 150 |
| lysoPC a C18:1 | Lipid, phospholipid | -0.32 | 0.028 | 0.118 | ↓ | -0.28 | 0.016 | 0.048 | ↓ | 86.7 | 69.5 | 71.5 |
| lysoPC a C18:0 | Lipid, phospholipid | -0.38 | 0.016 | 0.108 | ↓ | -0.28 | 0.047 | 0.057 | ↓ | 53.3 | 40.9 | 43.8 |
| PC ae C30:1 | Lipid, phospholipid | -0.42 | 0.016 | 0.108 | ↓ | -0.33 | 0.009 | 0.048 | ↓ | 0.17 | 0.12 | 0.13 |
| PC aa C40:1 | Lipid, phospholipid | -0.59 | 0.016 | 0.108 | ↓ | -0.48 | 0.047 | 0.057 | ↓ | 2.70 | 1.79 | 1.94 |
| lysoPC a C17:0 | Lipid, phospholipid | -0.68 | 0.009 | 0.108 | ↓ | -0.56 | 0.009 | 0.048 | ↓ | 1.48 | 0.93 | 1.01 |
| PC aa C42:1 | Lipid, phospholipid | -0.86 | 0.016 | 0.108 | ↓ | -0.87 | 0.028 | 0.048 | ↓ | 1.22 | 0.67 | 0.67 |
| **Liver** | | | | | | | | | | | | |
| Kynurenine | Amino acid | 2.12 | 0.009 | 0.011 | ↑ | 3.18 | 0.009 | 0.007 | ↑ | 13.1 | 57.2 | 119 |
| Citrulline | Amino acid | 1.45 | 0.009 | 0.011 | ↑ | 2.81 | 0.009 | 0.007 | ↑ | 1908 | 2506 | 5227 |
| AC C18 | Lipid, β-oxidation | 1.40 | 0.009 | 0.011 | ↑ | 2.75 | 0.009 | 0.007 | ↑ | 0.092 | 0.24 | 0.62 |
| Lysine | Amino acid | 0.90 | 0.009 | 0.011 | ↑ | 2.46 | 0.009 | 0.007 | ↑ | 1126 | 2095 | 6199 |
| Ornithine | Amino acid | 0.85 | 0.047 | 0.032 | ↑ | 2.45 | 0.009 | 0.007 | ↑ | 643 | 1156 | 3503 |
| Histamine | Amino acid | 1.25 | 0.028 | 0.023 | ↑ | 2.34 | 0.009 | 0.007 | ↑ | 0.34 | 0.81 | 1.74 |
| Valine | Amino acid | 0.58 | 0.028 | 0.023 | ↑ | 2.12 | 0.009 | 0.007 | ↑ | 2465 | 3693 | 10719 |
| Dopamine | Amino acid | 1.28 | 0.009 | 0.011 | ↑ | 2.09 | 0.009 | 0.007 | ↑ | 0.042 | 0.10 | 0.18 |
| AC C16 | Lipid, β-oxidation | 1.31 | 0.009 | 0.011 | ↑ | 2.07 | 0.009 | 0.007 | ↑ | 0.12 | 0.30 | 0.51 |
| AC C5:1 | Lipid, β-oxidation | 1.06 | 0.009 | 0.011 | ↑ | 1.96 | 0.009 | 0.007 | ↑ | 0.064 | 0.13 | 0.25 |
| Carnitine | Lipid, β-oxidation | 0.55 | 0.047 | 0.032 | ↑ | 1.84 | 0.009 | 0.007 | ↑ | 425 | 621 | 1521 |
| Glycine | Amino acid | 0.70 | 0.009 | 0.011 | ↑ | 1.71 | 0.009 | 0.007 | ↑ | 4550 | 7388 | 14854 |
| Methionine-Sulfoxide | Amino acid | 0.78 | 0.009 | 0.011 | ↑ | 1.68 | 0.009 | 0.007 | ↑ | 12.8 | 22.0 | 41.2 |
| AC C3:1 | Lipid, β-oxidation | 0.64 | 0.028 | 0.023 | ↑ | 1.33 | 0.009 | 0.007 | ↑ | 0.023 | 0.036 | 0.058 |

| Biomarker | Metabolism | Low dose group | | | | High dose group | | | | Concentration(pmol/mg wet) | | |
|---|---|---|---|---|---|---|---|---|---|---|---|---|
| | | FC[a] | p | q | | FC[a] | p | q | | Control | Low | High |
| cis-8,11,14-Eicosatrienoic acid | Polyunsaturated fatty acids | 0.43 | 0.028 | 0.023 | ↑ | 1.15 | 0.009 | 0.007 | ↑ | 234 | 315 | 518 |
| AC C8:1 | Lipid, β-oxidation | 0.54 | 0.009 | 0.011 | ↑ | 1.07 | 0.009 | 0.007 | ↑ | 0. 13 | 0. 19 | 0. 27 |
| PC ae C40:0 | Lipid, phospholipid | 0.42 | 0.009 | 0.011 | ↑ | 0.66 | 0.009 | 0.007 | ↑ | 96. 7 | 129 | 153 |
| linolenic acid | Polyunsaturated fatty acids | -1.00 | 0.016 | 0.015 | ↓ | -3.50 | 0.008 | 0.007 | ↓ | 30. 0 | 15. 0 | 2. 66 |

[a]: Fold change

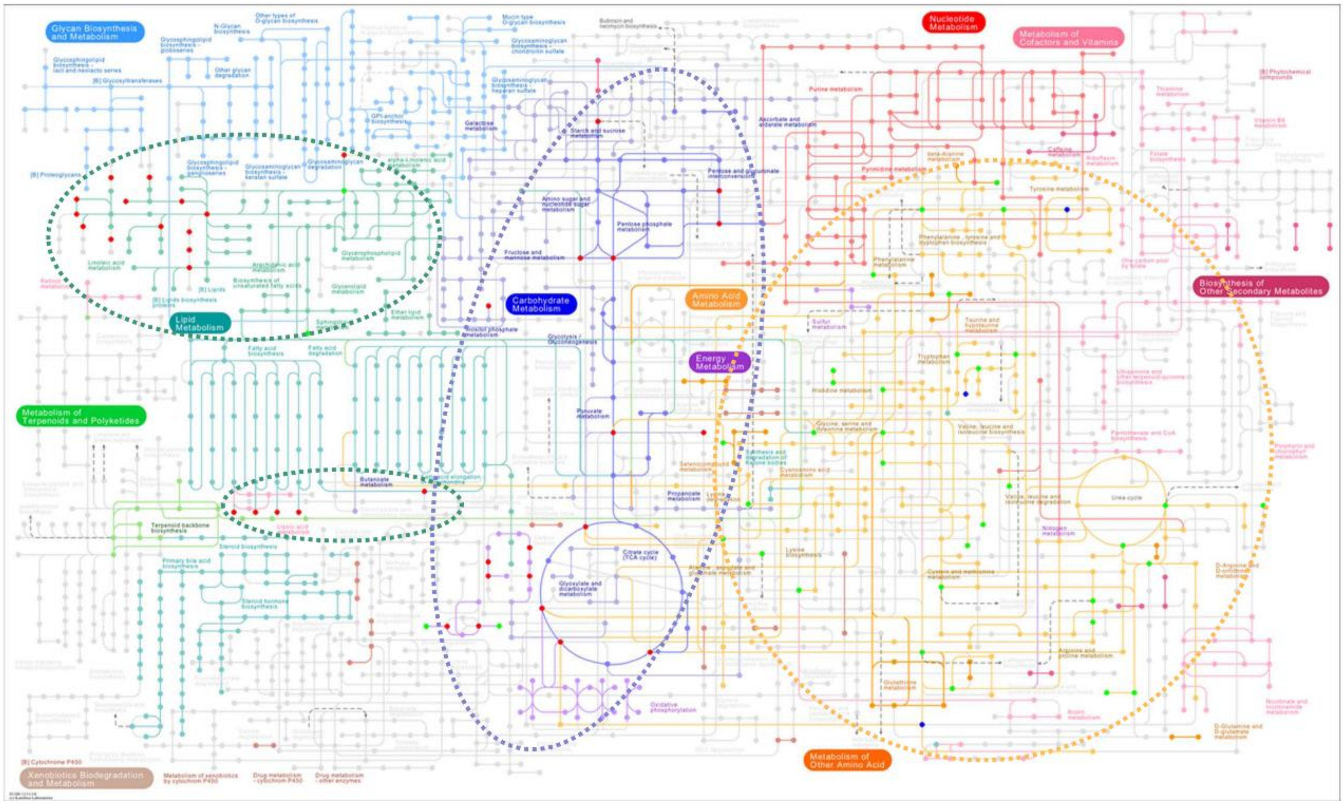


**Supplementary Figure S1. KEGG metabolic pathways mapping selected metabolites: blue: metabolites quantified only for brain; red: metabolites quantified only for liver; green: metabolites quantified for both brain and liver.**

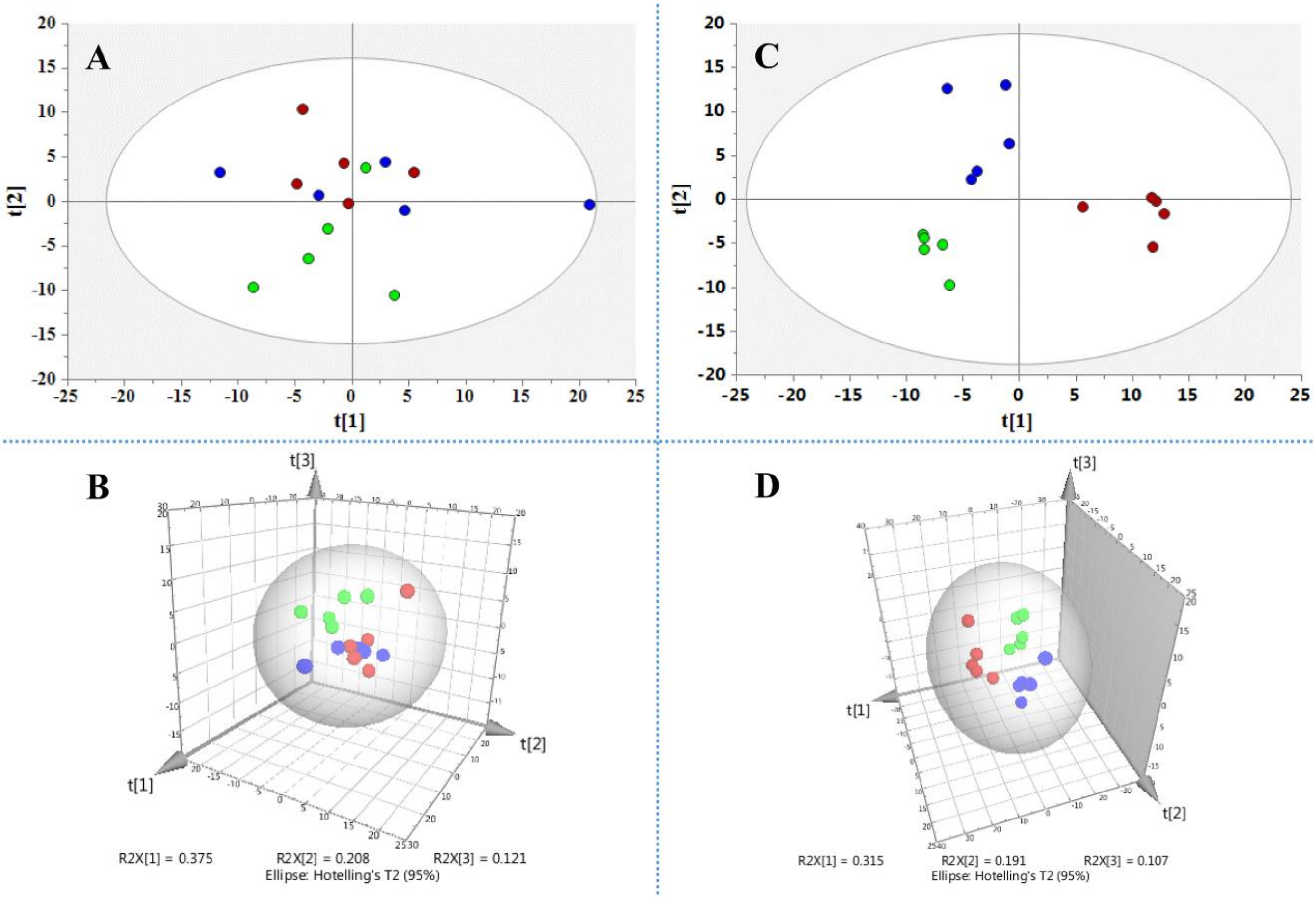


**Supplementary Figure S2. PCA score plots of metabolic profiles in brain (A and B) and liver (C and D). Panels A and C for the first two components and panels B and D for the first three components. Red for the high-dose group; Blue for the low-dose group; Green for the control group.**

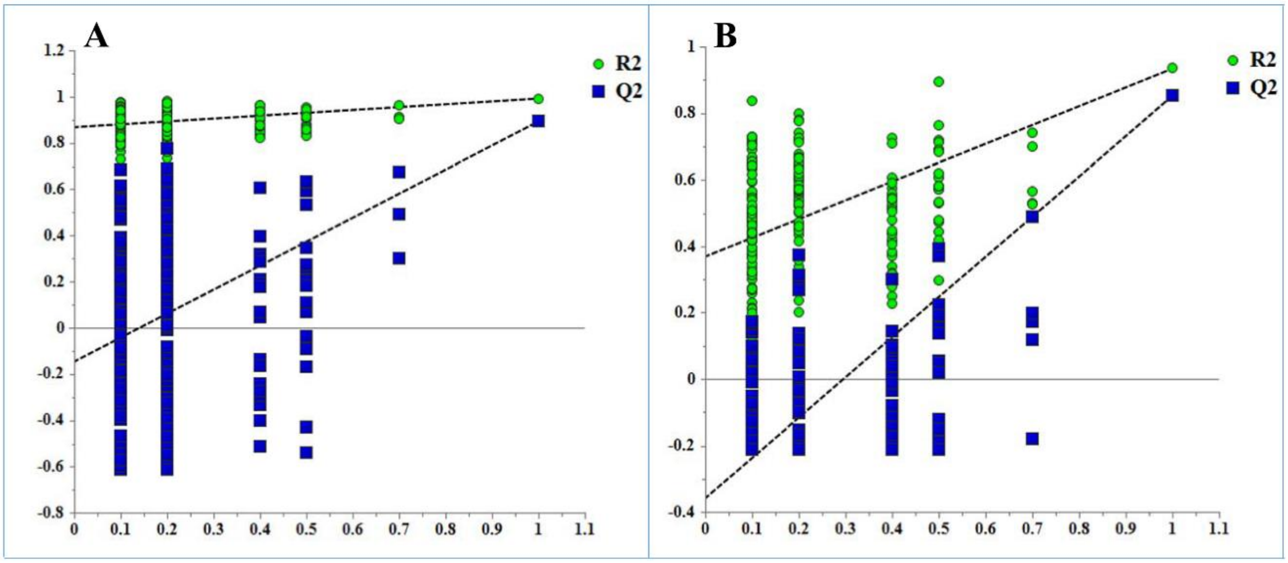


**Supplementary Figure S3. Permutations plot for PLS-DA models for brain (A) and liver (B).**

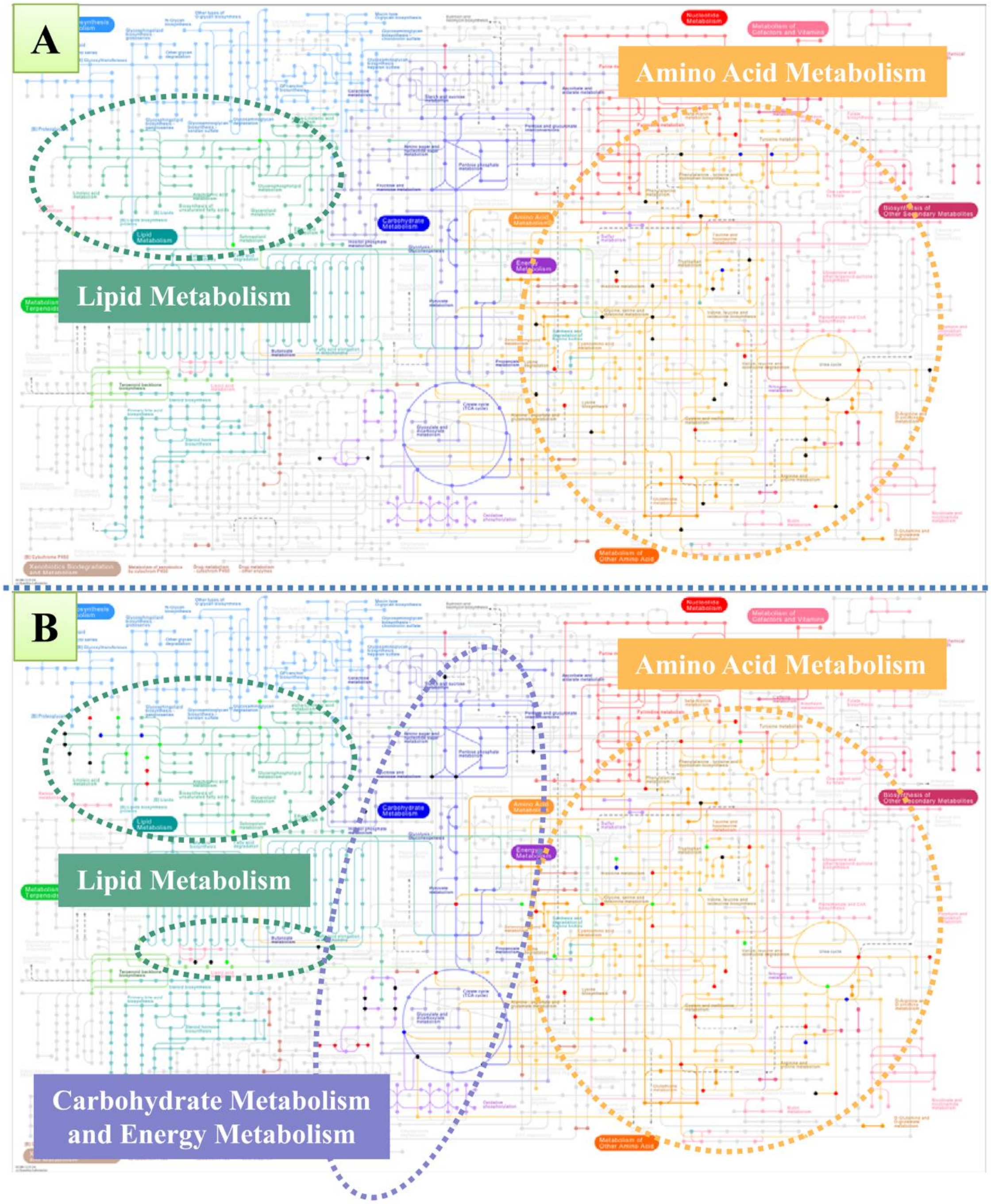


**Supplementary Figure S4. Metabolic pathway analysis. KEGG metabolic pathways mapping the detected metabolites in brain (A) and liver (B), red: differential metabolites only for high-dose group; blue: differential metabolites only for low-dose group; green: differential metabolites for both two-exposure groups.**

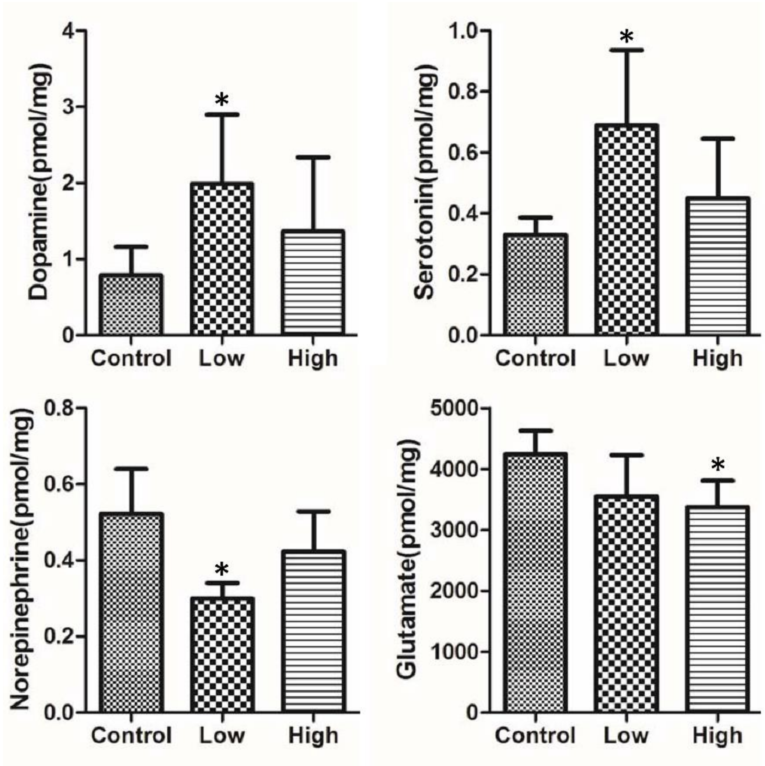


**Supplementary Figure S5. Mean concentrations of dopamine, serotonin, norepinephrine and glutamate in brain among three groups. *: $p < 0.05$; the confidence intervals are SD.**

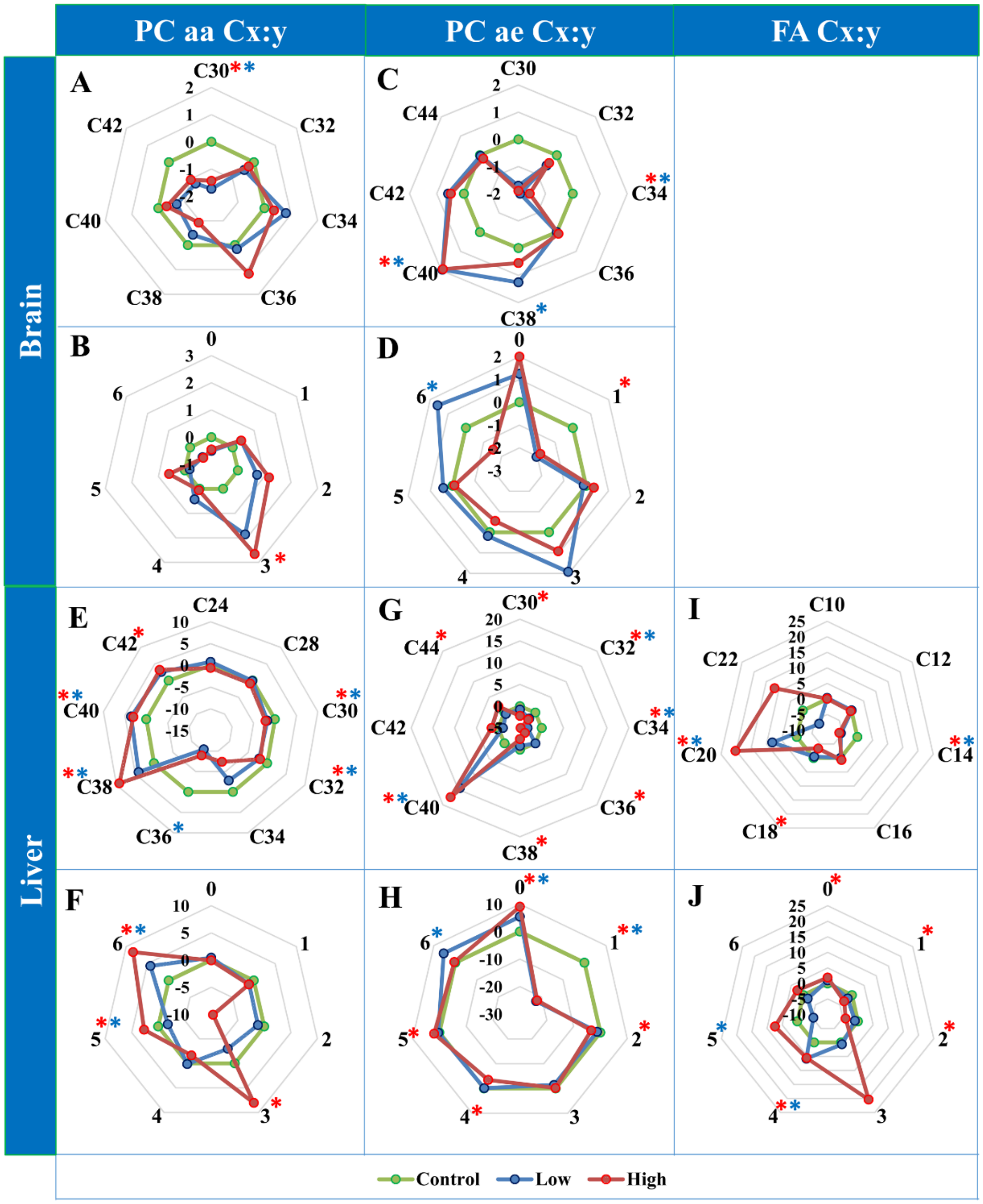


**Supplementary Figure S6. Z-score of phosphatidylcholines (PC aa Cx:y) and plasmalogen/plasmenogen phosphatidylcholines (PC ae Cx:y) in brain or liver, and Z-score of fatty acids (FA Cx:y) in liver. The panel A, C, E, G, and I show the profile of Z-score based on carbon atoms (Cx). The panel B, D, F, H, and J show the profile of Z-score based on double bonds (y). *: $p < 0.05$ (blue: between low-dose group and control group; red: between high-dose group and control group).**

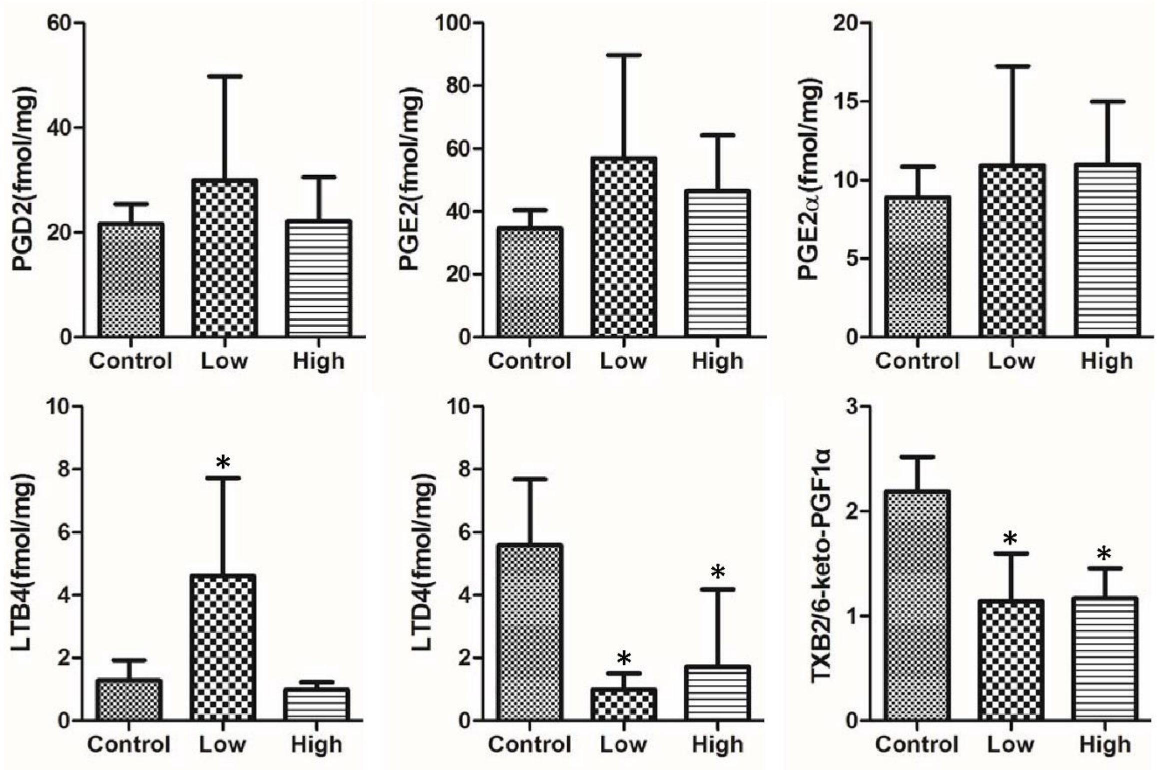


**Supplementary Figure S7. Mean concentrations of prostaglandins, thrombaxanes, and leukotrienes in liver among three groups. *: $p < 0.05$ ; the confidence intervals are SD.**
